# Pain management and related factor exploration of rheumatoid arthritis based on nursing science precision health model: a retrospective analysis of 287 cases

**DOI:** 10.3389/fmed.2026.1789003

**Published:** 2026-04-02

**Authors:** Hua Ren, Guohua Wei, Yuejin Li, Sanjiao Liu, Yifan Guo, Mengyang Zhang, Ziwei Kong

**Affiliations:** 1 School of Nursing, Shanxi University of Chinese Medicine, Taiyuan, Shanxi, China; 2 Department of Geriatrics, First Hospital of Shanxi Medical University, Taiyuan, Shanxi, China; 3 Third Clinical Medical School, Shanxi University of Chinese Medicine, Taiyuan, Shanxi, China

**Keywords:** biomarkers, nursing science precision health model, pain management, phenotype-based interventions, rheumatoid arthritis

## Abstract

**Introduction:**

Rheumatoid arthritis (RA) is a chronic inflammatory disease characterized by pain, functional disability, and comorbidities. Pain management in RA is complex due to both inflammatory and non-inflammatory mechanisms. The Nursing Science Precision Health (NSPH) model offers a personalized approach to pain management, integrating symptom measurement, phenotypic analysis, and biomarker data to guide tailored interventions.

**Methods:**

The recorded data of 287 RA patients were retrospectively archived and categorized into three pain phenotypes: inflammatory pain, non-inflammatory pain, and mixed pain. Pain was assessed using the Visual Analog Scale (VAS), and biomarkers were measured at baseline. Psychological factors, including anxiety, depression, and sleep quality, were also evaluated. Patients’ phenotype-specific interventions were extracted from clinical records: pharmacological treatment for inflammatory pain, psychological counseling and mindfulness-based stress reduction for non-inflammatory pain, and combined therapies for mixed pain. Follow-up assessments were conducted at 12 weeks.

**Results:**

Significant improvements were observed across all pain phenotypes. Inflammatory pain patients showed reductions in CRP, ESR, and VAS pain scores. Non-inflammatory pain patients experienced reductions in anxiety, depression, and VAS scores, with improvements in sleep quality. Mixed pain patients benefited from both pharmacological and psychological interventions. Patient-reported outcomes, including quality of life and functional status, improved significantly, with 82.6% expressing satisfaction with their pain management plan.

**Discussion:**

The NSPH model offers an effective framework for personalized RA pain management, demonstrating that phenotype-based interventions improve pain outcomes, reduce psychosocial distress, and enhance quality of life. This approach holds potential for broader application in chronic pain management and warrants further research to optimize its implementation.

## Introduction

Rheumatoid arthritis (RA) is a chronic autoimmune disease characterized by systemic inflammation, synovial proliferation, and progressive joint destruction, leading to substantial pain and functional impairment. The disease affects approximately 0.5–1% of the global population and remains a leading cause of disability worldwide, contributing substantially to years lived with disability and the healthcare burden. Pain remains one of the most prominent and disabling symptoms in RA, significantly affecting physical function, psychological wellbeing, and social participation (1, 2). Although advances in pharmacological therapies, including disease-modifying anti-rheumatic drugs (DMARDs) and biologics, have improved disease control, a considerable proportion of patients continue to experience persistent pain, highlighting the complexity of underlying mechanisms and the limitations of inflammation-centered treatment strategies (3–6).

Traditionally, RA pain has been attributed primarily to nociceptive processes driven by synovial inflammation and joint damage. However, this view is increasingly recognized as insufficient, as many patients report ongoing pain despite well-controlled inflammatory activity. Emerging evidence supports the presence of nociplastic pain mechanisms in RA, characterized by central sensitization and altered pain processing within the central nervous system (6–8). In addition, psychosocial factors such as anxiety, depression, and sleep disturbance contribute substantially to pain amplification and persistence. These factors not only exacerbate pain perception but also interfere with treatment adherence and recovery, forming a self-reinforcing cycle of symptom burden (9). Collectively, these findings indicate that RA pain arises from a complex interaction of inflammatory, neurobiological, and psychosocial processes, necessitating a multidimensional approach to assessment and management.

Despite growing recognition of this complexity, current RA management strategies remain largely focused on pharmacological and immunological interventions. While effective in controlling disease activity, these approaches often fail to address persistent pain driven by non-inflammatory mechanisms (1, 7). Increasing attention has therefore been directed toward nursing-led and multidisciplinary interventions that incorporate psychological support, lifestyle modification, and patient education. Such approaches have demonstrated improvements in treatment adherence, emotional wellbeing, and functional outcomes, particularly when delivered through structured and continuous care models (9–11). However, these interventions are frequently implemented without a unified theoretical framework, limiting their ability to guide individualized, mechanism-based care.

To address these limitations, precision health approaches have been proposed to integrate biological, behavioral, and environmental determinants of symptoms into personalized care strategies. The Nursing Science Precision Health (NSPH) model represents a structured framework for applying these principles in clinical practice. This model incorporates four key components: precise symptom measurement, phenotypic analysis considering lifestyle and environmental factors, biomarker discovery, and targeted intervention development. By transitioning care from protocol-driven to data-informed and adaptive approaches, the NSPH model enables the integration of clinical indicators, psychosocial profiles, and biological signals to support individualized symptom management (12, 13). Previous studies have demonstrated that applying this framework to chronic inflammatory pain can improve symptom control and patient engagement by aligning interventions with underlying mechanisms (13, 14).

From a biological perspective, accumulating evidence highlights the importance of biomarkers in understanding RA pain. Cytokines such as TNF-α, IL-6, and the Th17/Treg ratio are involved not only in inflammatory activity but also in pain modulation and sensitization (15). Emerging studies have also identified novel targets, such as P2 × 7R, as potential indicators of pain severity within precision health frameworks (16). In parallel, psychosocial factors remain critical determinants of pain experience. A substantial proportion of RA patients with severe pain exhibit anxiety or depressive symptoms, which can exacerbate disease burden and functional impairment through neuroimmune interactions (17, 18). Interventions targeting these factors, including cognitive-behavioral therapy and mindfulness-based approaches, have shown effectiveness in reducing pain and improving mental health outcomes (19). Together, these findings support the integration of biomarker assessment with psychosocial evaluation to achieve a more comprehensive understanding of RA pain.

Lifestyle factors, including dietary patterns, may also influence inflammation and symptom burden in RA, although existing evidence remains heterogeneous. While some studies suggest that exclusion diets may improve pain and inflammatory markers, these findings are limited and not universally applicable (20). In contrast, higher-quality evidence supports Mediterranean-style and plant-based diets, which are associated with reduced inflammation and improved patient-reported outcomes (21–25). These observations further emphasize the importance of individualized, phenotype-based approaches rather than uniform recommendations. Despite these advances, the NSPH model has rarely been systematically applied to guide nursing practice in RA pain management. This gap reflects a disconnect between expanding biomedical knowledge and its translation into individualized, bedside care. In particular, there remains a lack of integrated approaches that combine biomarker profiling, psychosocial assessment, and phenotype-based intervention strategies within a unified framework.

Therefore, the present study applies the NSPH model to RA pain management by integrating clinical, biological, and psychosocial data to develop phenotype-based, nurse-led interventions. Specifically, this study aims to (1) classify patients into inflammatory, non-inflammatory, and mixed pain phenotypes using multidimensional assessments; (2) incorporate immune, neuroendocrine, and psychosocial biomarkers into phenotype characterization; and (3) evaluate the effectiveness of phenotype-specific interventions. By operationalizing the precision health framework in a clinical context, this study seeks to improve individualized pain management and provide a foundation for more effective, mechanism-based care strategies in RA.

## Materials and methods

### Study design and participants

The current retrospective study included 287 patients with rheumatoid arthritis (RA), whose clinical information was collected from rheumatology clinics across five hospitals from Jan 2022 to Dec 2023. Data were obtained from existing medical records and institutional databases. The study utilized both quantitative and qualitative approaches, guided by the Nursing Science Precision Health (NSPH) model, to explore pain management strategies and related factors in RA patients.

Given the retrospective design, no formal sample size calculation was performed; instead, the sample size was determined by the total number of patients meeting the inclusion and exclusion criteria during the study period. Initially, a total of 356 medical records of patients with RA were initially screened from rheumatology clinics across five hospitals during the study period. After applying the inclusion and exclusion criteria, 69 patients were excluded, including those with incomplete clinical data (*n* = 13), coexisting autoimmune diseases (*n* = 16), active infections (*n* = 10), pregnancy or lactation (*n* = 17), and participation in other clinical trials (*n* = 13). Ultimately, 287 patients met all eligibility criteria and were included in the final analysis. Eligible patients were aged 18–65 years, had a confirmed diagnosis of RA based on the American College of Rheumatology criteria, and had documented pain intensity ≥ 4 on the Visual Analog Scale (VAS) within one week prior to baseline evaluation.

This study was conducted in accordance with the Declaration of Helsinki and was approved by the Institutional Review Boards of the participating hospitals (no. 2022012412). Given the retrospective design based on de-identified medical records, the requirement for written informed consent was waived by the ethics committees.

### Data collection

Data were collected to evaluate pain, psychosocial status, fatigue, sleep, functional status, and laboratory tests for RA biomarkers. Table 1 summarizes the multiple tools and laboratory assessments collected during this study. The validated Chinese versions of the different scales were used. All measures were collected at baseline and again at 12 weeks.

**TABLE 1:** Data collection instruments.

Variables	Collection methods
Pain measures	
Pain intensity: Visual Analog Scale (VAS)	Straight line, 10 cm in length, with one end representing “no pain” and the other end representing “worst pain imaginable.”
Overview of chronic pain experience and its impact on daily life: Global Pain Scale (GPS)	Self-administered questionnaire measuring pain intensity, emotional response to the pain, clinical impact of the pain, and activity limitations due to pain. A total of 33 items, measured on a 0–10 scale.
Psychosocial measures	
Hospital Anxiety and Depression Scale (HADS)	14-item self-report screening tool for anxiety and depression.
Social Support Rating Scale (SSRS)	Self-report questionnaire measuring the availability, adequacy, and perceived quality of social support.
Pain Catastrophizing Scale (PCS)	Self-report questionnaire measuring catastrophizing (an exaggerated negative orientation toward noxious stimuli) 0.13 items scored on a 5-point Likert scale with total scores ranging from 0 to 52. Scores above 30 indicate clinically significant levels of catastrophizing.
Short Form-36 (SF-36)	Self-report questionnaire. Assesses overall health status and quality of life. A total of 36 items and eight subscales. Scores range from 0 to 100 with lower scores indicating more disability.
Fatigue and sleep	
FACIT-Fatigue Scale (FACIT-F)	Self-report questionnaire used to measure an individual’s level of fatigue during their usual daily activities over the past week. A total of 13 items measured on a four-point Likert scale of 0–4. Scores range from 0 to 52, with higher scores indicating greater fatigue.
Pittsburgh Sleep Quality Index (PSQI)	Self-report questionnaire used to evaluate sleep quality. A total of 10 items (some multicomponent) with total scores ranging from 0 (better sleep quality) to 21 (worse sleep quality).
Clinical and functional assessments	
Demographic and clinical data	Age, sex, disease duration, comorbidities, medication history
Health Assessment Questionnaire (HAQ)	Self-report questionnaire to evaluate functional ability and disability levels. A total of 20 items rated on a scale from 0 (no difficulty) to 3 (unable to do). The higher the score the greater the disability.
Disease activity score 28	A four-component composite measure used to assess RA activity. Component one is an evaluation of 28 specific joints (shoulders, elbows, wrists, metacarpophalangeal, proximal interphalangeal, knees) for tenderness. The number of tender joints is recorded. The second part is the number of swollen joints of the same 28. The third component is a laboratory measure of inflammatory markers—Erythrocyte sedimentation rate (ESR) and C-reactive protein (CRP) levels. The fourth component is a patient’s self-assessed health status, measured with the patient global assessment (PGA) of disease activity on a visual analog scale. A formula is used to arrive at total scores. Scores less than 2.6 indicate remission, scores less than 3.2 mean low disease activity, scores between 3.2 and 5.1 suggest moderate disease activity, and scores greater than 5.1 indicate high disease activity.
Biomarker assessments	
ESR: erythrocyte sedimentation rate	Marker for inflammatory response. High with RA.
CRP: C-reactive protein	Marker for inflammatory response. High with RA.
RF: rheumatoid factor	Autoantibody produced against connective tissues in joints. Present in most people with RA
Anti-CCP: anti-cyclic citrullinated peptide	Autoantibody that can be detected at an early stage of RA.
Cytokines TNF-α, IL-2 IL-6, IL-17A, IL-10	Small proteins that mediate and regulate the immune and inflammatory response
Th17 and Treg cell counts	Biomarkers in RA management and treatment that indicate inflammation and joint damage.
Th17/Treg ratio	An autoimmune balance index between inflammatory TH17 cells and regulatory Tregs. The lower the ratio, the less inflammation.
SP (Substance P) and CGRP (calcitonin gene-related peptide)	Substance P is a neuropeptide involved in neurogenic inflammation of RA. CRGP has a pro-inflammatory role in RA
ACTH (adrenocorticotropic hormone) and Serum Cortisol	Measures the HPA (hypothalamic-pituitary-adrenal) axis response to stress. Plays a role in pain management by regulating the body’s response to stress. Chronic stress can lead to prolonged activation of the HAP axis, potentially contributing to the inflammatory processes associated with RA
MDA: malondialdehyde SOD: superoxide dismutase GSH-Px: glutathione peroxidase	Biomarkers for oxidative stress. Oxidative stress plays a central role in the pathology of RA by producing reactive oxygen species that damage joint tissues and exacerbate inflammation and tissue injury MDA is elevated in RA patients, correlating positively with disease activity. SOD is significantly decreased in RA GSH-Px is often reduced in RA

### Interventions and follow-up

**FIGURE 1:**
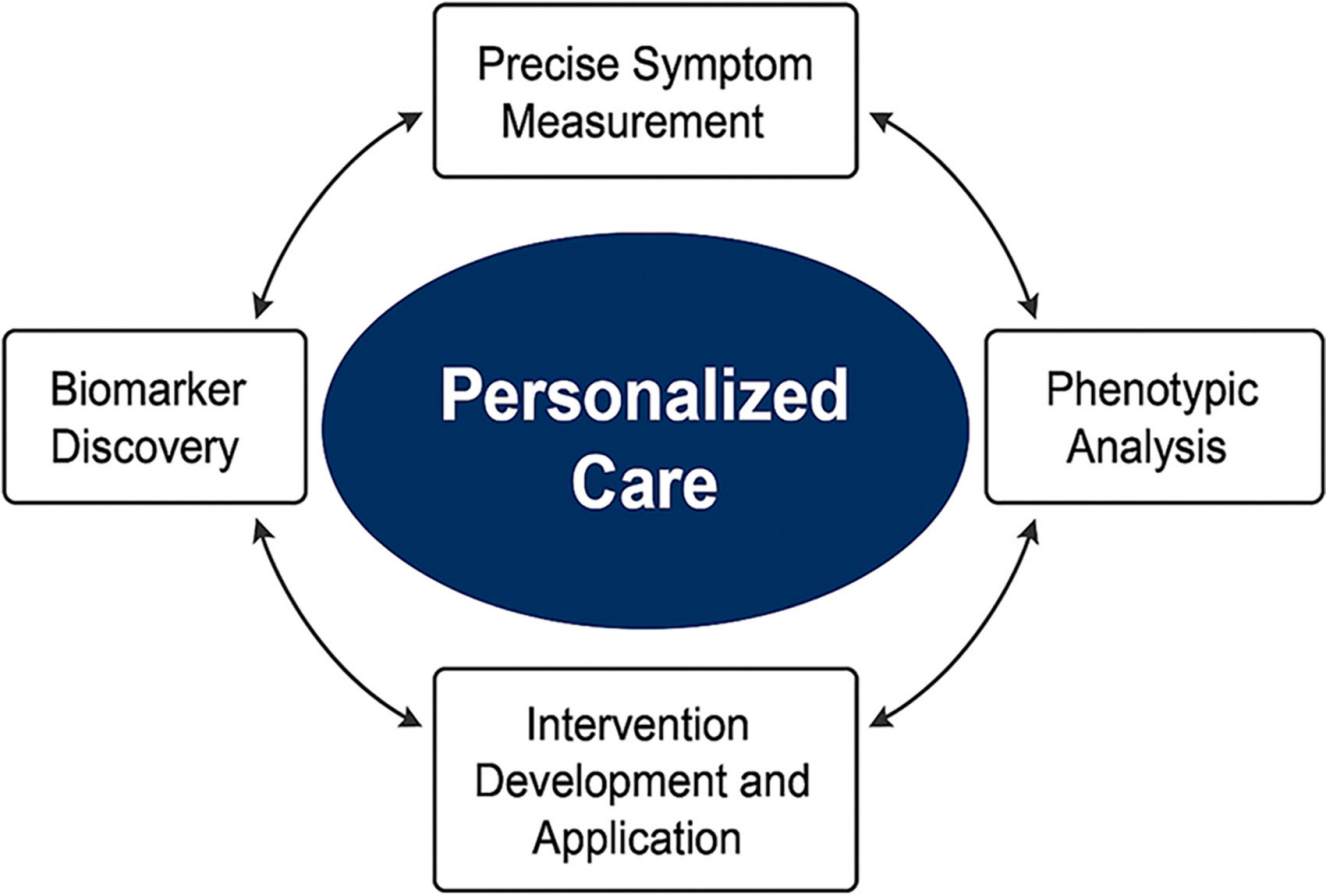
Conceptual framework of the Nursing Science Precision Health (NSPH) model applied to rheumatoid arthritis (RA) pain management.

Interventions were designed according to the NSPH model, emphasizing individualized, phenotype-based management (Figure 1). Briefly, the NSPH model was developed to bridge precision medicine and nursing science by emphasizing individualized symptom assessment, data integration, and adaptive care. It operates through an iterative, four-component process that connects discovery science with clinical decision-making. Precise symptom measurement (Component A) uses validated instruments and digital tools to quantify patient-reported outcomes and physiological parameters with multi-domain, repeated time-point longitudinal symptom assessment. Phenotype characterization (Component B) integrates symptom clusters, lifestyle, environmental exposure, and psychosocial patterns to define distinct patient phenotypes. Biomarker discovery (Component C) identifies molecular, immune, or neuroendocrine indicators that differentiate phenotypes or predict responses. Targeted intervention and evaluation (Component D) designs and continuously refines interventions based on phenotype-specific mechanisms, feeding results back into the measurement loop for precision improvement. Each intervention strategy targeted both biological and psychosocial determinants of pain, ensuring comprehensive coverage across inflammatory, non-inflammatory, and mixed pain phenotypes. Based on the aforementioned criteria, the total of 287 patients were categorized into inflammatory (*n* = 111), non-inflammatory (*n* = 85), and mixed pain phenotypes (*n* = 91).

#### Inflammatory pain interventions

Patients with inflammatory pain received pharmacological therapies to control inflammation and disease activity. Conventional disease-modifying anti-rheumatic drugs (DMARDs) (e.g., methotrexate, sulfasalazine) and biologic agents inhibiting TNF-α or IL-6 (e.g., Enbrel, Remicade, Actemra) were prescribed as indicated to control inflammation. NSAIDs and corticosteroids were administered for short-term relief during flare-ups. Treatment response was closely monitored using the DAS28 and biomarker tracking (CRP, ESR, cytokines, and Th17/Treg balance) to ensure adequate suppression of inflammation. No formal psychosocial interventions (e.g., psychological counseling, mindfulness-based stress reduction, or cognitive-behavioral strategies) were delivered to this phenotype.

#### Non-inflammatory pain interventions

Patients with non-inflammatory pain underwent interventions focused on psychosocial, neuroendocrine, and lifestyle factors. Cognitive Behavioral Therapy (CBT) and Mindfulness-Based Stress Reduction (MBSR) were delivered to reduce catastrophizing, anxiety, and depression. Fatigue was addressed using structured activity-rest cycles guided by FACIT-Fatigue results, while dietary interventions emphasized anti-inflammatory nutrition with reduced gluten, red meat, and dairy, and increased intake of omega-3 fatty acids and antioxidants. Acupuncture without trigger point therapy was provided twice weekly as a complementary therapy to alleviate pain and improve sleep quality.

#### Mixed pain interventions

Patients with mixed pain received an integrated approach combining pharmacological, psychological, and complementary strategies. Pharmacological treatment targeted inflammatory components with DMARDs or biologics, while CBT, MBSR, and sleep hygiene strategies addressed central sensitization and psychological distress. Acupuncture combined with trigger point therapy was used to reduce neuromuscular pain and improve joint mobility, providing a comprehensive management strategy for this subgroup. Acupuncture was administered twice weekly, and trigger point therapy was delivered once weekly as an additional modality to address the neuromuscular component of pain. These phenotype-specific differences were prespecified because non-inflammatory pain management emphasized psychosocial and behavioral drivers, whereas mixed pain required simultaneous targeting of inflammatory mechanisms and neuromuscular pain features.

#### Follow-up and evaluation

Follow-up assessments were performed at 12 weeks to evaluate treatment effectiveness across all dimensions. Outcomes were evaluated across four domains: (1) clinical disease activity, (2) biomarker modulation, (3) psychosocial status, and (4) patient-reported functional and quality-of-life measures as follows.

Pain outcomes were reassessed with the VAS and GPS, while psychosocial outcomes included the HADS, PSQI, SSRS, FACIT-Fatigue, PCS, and SF-36. Clinical outcomes were monitored using the HAQ, DAS28, swollen/tender joint counts, and PGA. Laboratory reassessments included ESR, CRP, RF, anti-CCP, cytokines (IL-2, IL-6, TNF-α, IL-17A, IL-10), Th17/Treg ratio, neuropeptides (Substance P, CGRP), endocrine markers (cortisol, ACTH), and oxidative stress indicators (MDA, SOD, GSH-Px). This multidimensional follow-up ensured comprehensive evaluation of both clinical improvements and broader biological and psychosocial adaptations across all pain phenotypes.

### Statistical analysis

All data were analyzed using SPSS version 25.0 (IBM Corp., Armonk, NY, USA). Descriptive statistics were used to summarize demographic characteristics, clinical parameters, psychosocial scores, and biomarker levels. Continuous variables were expressed as mean ± standard deviation (SD) or median (interquartile range), depending on distribution, while categorical variables were presented as frequencies and percentages. Group differences between pain phenotypes (inflammatory, non-inflammatory, and mixed) were assessed using one-way ANOVA or Kruskal–Wallis tests for continuous variables and chi-square tests for categorical variables. To evaluate intervention effects, paired *t*-tests or Wilcoxon signed-rank tests were applied to compare baseline and follow-up values at 12 weeks. Between-group changes were examined using repeated-measures ANOVA with Bonferroni correction. Correlation analyses (Pearson or Spearman) were performed to explore relationships between pain intensity (VAS, GPS) and explanatory factors including biomarkers (CRP, ESR, IL-2, IL-6, TNF-α, IL-17A, IL-10, Th17/Treg ratio, neuropeptides, oxidative stress markers, cortisol, ACTH), psychosocial measures (HADS, PSQI, SSRS, FACIT-F, PCS, SF-36), and clinical indices (HAQ, DAS28, PGA). Multivariable logistic regression was conducted to identify independent predictors of high pain (VAS ≥ 6) and poor quality of life (SF-36 < median). To further investigate mechanistic pathways, structural equation modeling (SEM) was applied to test hypothesized associations between inflammatory activity, psychosocial distress, central sensitization markers, and patient-reported outcomes. Goodness-of-fit indices (χ^2^/df, RMSEA, CFI, TLI) were used to evaluate model adequacy. For all analyses, a two-tailed *P*-value < 0.05 was considered statistically significant.

## Results

### Baseline clinical, immunological, and psychosocial characteristics of the cohort

A total of 287 patients with rheumatoid arthritis (RA) were included in the study. Baseline characteristics are summarized in Table 2. The mean CRP was 16.4 ± 8.9 mg/L, and ESR was 36.5 ± 14.3 mm/h, indicating moderate to high systemic inflammatory activity at baseline. A majority of patients were RF-positive (72.4%) and anti-CCP-positive (68.3%), reflecting a predominantly seropositive RA population with established autoimmune features. Among cytokines, IL-6 levels averaged 32.6 ± 11.2 pg/mL, TNF-α 24.7 ± 9.6 pg/mL, IL-2 18.9 ± 7.3 pg/mL, IL-17A 21.4 ± 8.6 pg/mL, while IL-10 averaged 9.8 ± 3.5 pg/mL. This cytokine profile is characterized by elevated pro-inflammatory mediators alongside relatively lower anti-inflammatory IL-10, suggesting a pro-inflammatory immune milieu. The mean Th17/Treg ratio was 2.5 ± 0.9, indicating a shift toward pro-inflammatory Th17 dominance and immune regulatory imbalance that is associated with persistent inflammation, heightened pain sensitivity, and reduced resolution capacity, consistent with RA immunopathology (Table 2).

**TABLE 2:** Baseline information for all patients (*n* = 287).

Variable	Value (mean ± SD)
C-reactive protein (CRP) (mg/L)	16.4 ± 8.9
Erythrocyte sedimentation rate (ESR) (mm/h)	36.5 ± 14.3
Rheumatoid factor (RF) positive (%)	72.4
Anti-CCP positive (%)	68.3
IL-6 (pg/mL)	32.6 ± 11.2
TNF-α (pg/mL)	24.7 ± 9.6
IL-2 (pg/mL)	18.9 ± 7.3
IL-17A (pg/mL)	21.4 ± 8.6
IL-10 (pg/mL)	9.8 ± 3.5
Th17/Treg ratio	2.5 ± 0.9
Substance P (pg/mL)	185 ± 56
CGRP (pg/mL)	142 ± 48
Cortisol (μg/dL)	11.8 ± 4.2
ACTH (pg/mL)	32.5 ± 11.4
MDA (nmol/mL)	5.2 ± 1.8
SOD (U/mL)	108 ± 24
GSH-Px (U/mL)	320 ± 65
HADS-anxiety (score)	11.2 ± 3.8
HADS-depression (score)	9.5 ± 3.4
PSQI (score)	9.8 ± 3.6
FACIT-Fatigue (score)	28.4 ± 8.2
PCS (score)	21.3 ± 7.9
SF-36 (score)	52.7 ± 12.6
HAQ (score)	1.9 ± 0.5
DAS28 (score)	4.9 ± 1.2
PGA (0–100)	61.4 ± 15.8

Neuropeptides and endocrine markers showed moderate elevations, with Substance P averaging 185 ± 56 pg/mL, and CGRP averaging 142 ± 48 pg/mL, suggesting enhanced nociceptive signaling and involvement of central pain amplification mechanisms. Cortisol (11.8 ± 4.2 μg/dL) and ACTH (32.5 ± 11.4 pg/mL) levels were within mid-range values, indicating partial activation but not exhaustion of the hypothalamic–pituitary–adrenal axis. Oxidative stress markers revealed increased lipid peroxidation (MDA 5.2 ± 1.8 nmol/mL), while antioxidant capacity (SOD 108 ± 24 U/mL, GSH-Px 320 ± 65 U/mL) remained relatively preserved, suggesting oxidative stress burden with partially maintained compensatory antioxidant defenses (Table 2). Psychosocial scores showed significant symptom burden: HADS-anxiety 11.2 ± 3.8, HADS-depression 9.5 ± 3.4, PSQI 9.8 ± 3.6, FACIT-F 28.4 ± 8.2, PCS 21.3 ± 7.9, and SF-36 52.7 ± 12.6. These scores indicate clinically relevant anxiety, depressive symptoms, sleep disturbance, fatigue, and reduced quality of life. Functional impairment was also evident (HAQ 1.9 ± 0.5, DAS28 4.9 ± 1.2, PGA 61.4 ± 15.8), consistent with active disease and significant patient-perceived disease burden (Table 2). Collectively, these findings indicate that the cohort presented with concurrent inflammatory activity, immune dysregulation, pain sensitization, and psychosocial distress at baseline.

### Distribution of pain phenotypes and their distinct biological and psychosocial profiles

Patients were categorized into inflammatory pain (38.7%), non-inflammatory pain (29.6%), and mixed pain (31.7%) groups, demonstrating substantial heterogeneity in pain mechanisms within the RA population (Table 3). VAS scores were highest in the non-inflammatory group (7.8 ± 1.6), followed by mixed (7.3 ± 1.5) and inflammatory (6.5 ± 1.7) groups, indicating that higher pain intensity was not exclusively associated with inflammatory burden. Inflammatory biomarkers were most elevated in the inflammatory phenotype (CRP 22.3 ± 7.4 mg/L, ESR 43.7 ± 15.8 mm/h, IL-6 38.2 ± 9.5 pg/mL, IL-17A 25.1 ± 7.6 pg/mL), reflecting inflammation-driven nociceptive pain mechanisms. In contrast, the non-inflammatory group exhibited lower inflammatory markers but worse psychosocial outcomes, suggesting pain predominantly mediated by central sensitization and psychological factors rather than peripheral inflammation (Table 3).

**TABLE 3:** Pain phenotypes and contributing factors.

Variable	Inflammatory pain (*n* = 111)	Non-inflammatory pain (*n* = 85)	Mixed pain (*n* = 91)
VAS (mean ± SD)	6.5 ± 1.7	7.8 ± 1.6	7.3 ± 1.5
CRP (mg/L)	22.3 ± 7.4	5.6 ± 3.8	12.8 ± 5.6
ESR (mm/h)	43.7 ± 15.8	12.6 ± 4.5	28.5 ± 12.4
RF (%)	84.5	57.4	67.8
Anti-CCP (%)	77.1	48.9	62.4
IL-6 (pg/mL)	38.2 ± 9.5	20.5 ± 5.9	34.6 ± 10.7
TNF-α (pg/mL)	26.7 ± 8.3	26.8 ± 7.2	25.6 ± 8.9
IL-2 (pg/mL)	19.4 ± 6.8	18.3 ± 5.7	20.1 ± 7.4
IL-17A (pg/mL)	25.1 ± 7.6	15.4 ± 5.1	21.8 ± 6.7
IL-10 (pg/mL)	7.1 ± 2.4	11.2 ± 3.6	9.3 ± 2.9
Th17/Treg ratio	3.1 ± 1.1	1.8 ± 0.7	2.6 ± 0.9
Substance P (pg/mL)	215 ± 64	158 ± 45	189 ± 52
CGRP (pg/mL)	161 ± 49	122 ± 36	145 ± 41
Cortisol (μg/dL)	9.7 ± 3.6	13.4 ± 4.2	11.2 ± 3.9
ACTH (pg/mL)	38.6 ± 12.8	29.4 ± 10.7	34.1 ± 11.6
MDA (nmol/mL)	6.1 ± 2.1	4.2 ± 1.6	5.1 ± 1.8
SOD (U/mL)	95 ± 21	121 ± 26	108 ± 23
GSH-Px (U/mL)	281 ± 58	346 ± 71	318 ± 65
HADS-anxiety	12.8 ± 3.4	15.6 ± 4.1	14.2 ± 3.7
HADS-depression	11.5 ± 3.2	14.1 ± 3.8	12.7 ± 3.5
PSQI	11.2 ± 3.6	13.4 ± 3.9	12.1 ± 3.8
FACIT-F	23.8 ± 7.1	19.5 ± 6.3	21.7 ± 6.7
PCS	26.4 ± 8.2	32.5 ± 9.1	29.1 ± 8.7
SF-36	47.2 ± 11.4	38.6 ± 10.7	42.1 ± 11.2

Specifically, non-inflammatory patients reported the highest HADS-anxiety (15.6 ± 4.1) and HADS-depression (14.1 ± 3.8) scores, along with poorer sleep quality (PSQI 13.4 ± 3.9) and greater catastrophizing (PCS 32.5 ± 9.1), indicating pronounced emotional and cognitive contributors to pain perception. The mixed phenotype demonstrated intermediate biomarker and psychosocial profiles, consistent with overlapping inflammatory and non-inflammatory pain mechanisms. Oxidative stress was most prominent in inflammatory pain (MDA 6.1 ± 2.1 nmol/mL), while antioxidant activity (SOD, GSH-Px) was relatively higher in non-inflammatory pain, suggesting differential metabolic and oxidative contributions across pain phenotypes (Table 3).

### Phenotype-specific treatment responses

Changes following phenotype-specific interventions are shown in Table 4. In patients with inflammatory pain, inflammatory markers showed the greatest reductions, with CRP decreasing by −12.5 ± 4.3 mg/L and ESR by −19.6 ± 6.8 mm/h, indicating effective suppression of systemic inflammation. Neuropeptides decreased significantly, with Substance P and CGRP reductions observed across groups, indicating alleviation of pain sensitization pathways. Endocrine markers (cortisol and ACTH) increased modestly post-intervention, suggesting partial normalization of HPA axis function (Table 4).

**TABLE 4:** Outcomes of different intervention strategies on changes in different parameters.

Variable	Inflammatory pain (*n* = 111)	Non-inflammatory pain (*n* = 85)	Mixed pain (*n* = 91)
Δ VAS	−2.3 ± 0.7*	−2.8 ± 1.1*	−2.5 ± 0.9*
Δ CRP (mg/L)	−12.5 ± 4.3*	−	−8.7 ± 3.6*
Δ ESR (mm/h)	−19.6 ± 6.8*	−	−12.3 ± 5.2*
Δ IL-6 (pg/mL)	−13.2 ± 5.4*	−	−10.4 ± 4.7*
Δ TNF-α (pg/mL)	−9.6 ± 3.1*	−7.2 ± 2.4*	−8.4 ± 2.9*
Δ IL-17A (pg/mL)	−10.5 ± 3.8*	−	−8.7 ± 3.2*
Δ IL-10 (pg/mL)	+2.1 ± 1.1*	+2.7 ± 1.3*	+2.3 ± 1.2*
Δ Th17/Treg ratio	−1.1 ± 0.5*	−	−0.8 ± 0.4*
Δ Substance P (pg/mL)	−41 ± 15*	−28 ± 11*	−36 ± 13*
Δ CGRP (pg/mL)	−32 ± 12*	−21 ± 9*	−27 ± 10*
Δ Cortisol (μg/dL)	+2.3 ± 1.2*	+1.8 ± 1.1*	+2.0 ± 1.1*
Δ ACTH (pg/mL)	+6.7 ± 3.4*	+4.5 ± 2.9*	+5.6 ± 3.1*
Δ MDA (nmol/mL)	−2.1 ± 0.8*	−1.4 ± 0.6*	−1.8 ± 0.7*
Δ SOD (U/mL)	+21 ± 9*	+18 ± 8*	+19 ± 8*
Δ GSH-Px (U/mL)	+62 ± 21*	+55 ± 19*	+58 ± 20*
Δ HADS-Anxiety	−5.2 ± 1.8*	−4.1 ± 1.3*	−3.9 ± 1.2*
Δ HADS-depression	−4.7 ± 1.6*	−3.6 ± 1.4*	−3.4 ± 1.2*
Δ PSQI	−3.8 ± 1.3*	−3.7 ± 1.2*	−3.2 ± 1.1*
Δ FACIT-F	+6.1 ± 2.4*	+7.2 ± 2.8*	+6.7 ± 2.6*
Δ PCS	−7.2 ± 2.6*	−8.4 ± 2.9*	−7.8 ± 2.7*
Δ SF-36	+11.5 ± 3.7*	+9.6 ± 3.4*	+10.2 ± 3.5*

*indicates a statistically significant within-group change (*p* < 0.05). –, parameters that were not applicable or not targeted for intervention within that specific phenotype. Only outcomes relevant to phenotype-specific interventions were analyzed.

Oxidative stress improved across all groups, with reductions in MDA and increases in antioxidant enzymes, indicating improved redox balance following intervention. Psychosocial improvements were substantial, with significant reductions in anxiety, depression, sleep disturbance, fatigue, and catastrophizing, alongside improved quality of life, demonstrating broad benefits extending beyond inflammatory control (*P* < 0.05).

### Patient-reported outcomes demonstrate significant functional gains and satisfaction

Patient-reported outcomes are summarized in Table 5. Functional status improved significantly, with HAQ scores decreasing by −0.5 to −0.7 points, representing clinically meaningful improvements in daily functioning (*P* < 0.05). Disease activity (DAS28) declined most markedly in inflammatory pain, consistent with inflammation-targeted therapeutic effects, while mixed and non-inflammatory groups also showed significant improvements (Table 5).

**TABLE 5:** Patient-reported outcomes of different intervention strategies.

Outcome	Inflammatory pain (*n* = 111)	Non-inflammatory pain (*n* = 85)	Mixed pain (*n* = 91)
HAQ score (baseline)	1.9 ± 0.5	1.8 ± 0.6	1.9 ± 0.5
HAQ score (12 weeks)	1.2 ± 0.3	1.3 ± 0.4	1.4 ± 0.3
Δ HAQ score	−0.7 ± 0.2*	−0.5 ± 0.2*	−0.5 ± 0.2*
DAS28 (baseline)	5.8 ± 1.3	5.4 ± 1.2	5.6 ± 1.2
DAS28 (12 weeks)	3.7 ± 0.9	3.9 ± 1.0	4.0 ± 0.9
Δ DAS28	−2.1 ± 0.6*	−1.5 ± 0.5*	−1.6 ± 0.5*
SF-36 (baseline)	47.2 ± 11.4	38.6 ± 10.7	42.1 ± 11.2
SF-36 (12 weeks)	58.7 ± 12.1	48.2 ± 11.4	52.3 ± 12.0
Δ SF-36	+11.5 ± 3.7*	+9.6 ± 3.4*	+10.2 ± 3.5*
Patient satisfaction (baseline %)	81.4	84.7	83.2
Patient satisfaction (12 weeks %)	89.7	87.5	86.8
Δ Patient satisfaction (%)	+8.3*	+2.8*	+3.6*

*P < 0.05 vs. baseline.

Quality of life improved across all phenotypes, with SF-36 increases of approximately 10 points, reflecting broad physical and mental health benefits. Patient satisfaction increased, particularly in the inflammatory group, indicating favorable patient-perceived effectiveness of phenotype-based management.

### Predictive value of biomarkers and psychosocial factors for pain phenotypes

Comprehensive regression analyses revealed that both inflammatory and psychosocial variables significantly contributed to pain severity and phenotype differentiation in patients with RA. Among inflammatory biomarkers, CRP, ESR, IL-6, and TNF-α levels were independently associated with the inflammatory-pain phenotype, indicating their utility as biological indicators of inflammation-driven pain (β = 0.28–0.36, *P* < 0.01). Anxiety (HADS-A) and depression (HADS-D) scores were significant predictors of pain persistence (β = 0.31 and 0.29, respectively; both *P* < 0.001), and poor sleep quality (PSQI > 8) showed a moderate correlation with higher VAS scores (*r* = 0.42, *P* < 0.001), highlighting psychosocial dysregulation as a major contributor to sustained pain burden. Nevertheless, social support (SSRS) demonstrated a protective effect (β = −0.25, *P* = 0.004), suggesting a mitigating role against pain severity. When all variables were entered into a multivariate model, biomarkers accounted for 42.5% and psychosocial factors for 37.8% of total variance in pain outcomes (adjusted R^2^ = 0.68). These findings indicate that both biological inflammation and psychological dysregulation independently contribute to the pain burden, reinforcing the need for integrated, precision-health nursing interventions targeting multidimensional mechanisms of pain.

### Cytokine modulation differs across pain phenotypes after intervention

**FIGURE 2:**
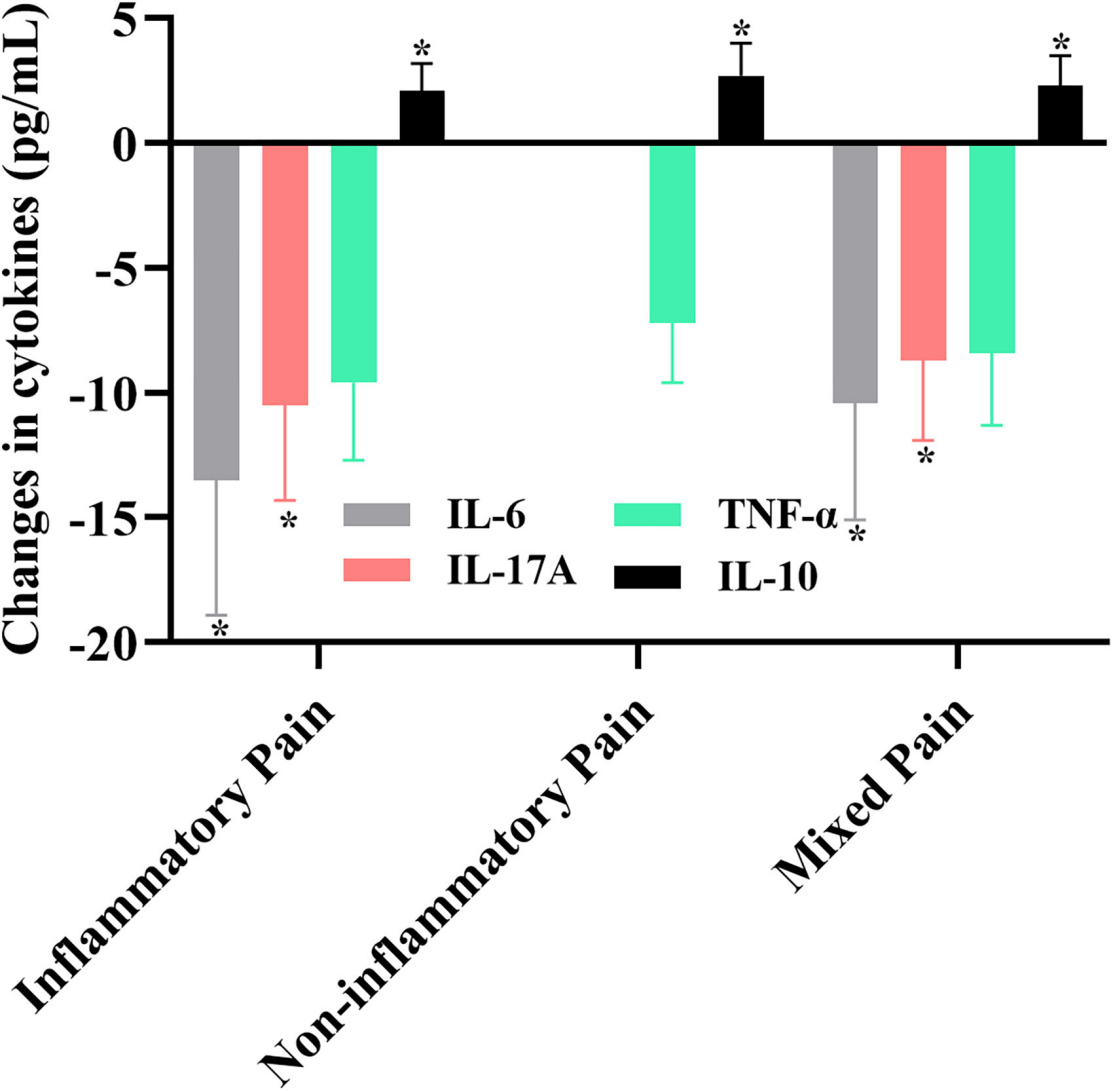
Changes in IL-6, IL-17A, TNF-α, and IL-10 across the three pain phenotypes after intervention (mean ± SD). *P < 0.05 vs. baseline.

Figure 2 illustrates changes in IL-6, IL-17A, TNF-α, and IL-10 across the three pain phenotypes after intervention. Significant reductions were observed in IL-6 and IL-17A in the inflammatory and mixed pain groups (*P* < 0.05), indicating phenotype-specific responsiveness of inflammatory pathways. In contrast, TNF-α reductions did not reach statistical significance after the between-group comparisons, suggesting a limited role for TNF-α modulation across phenotypes. IL-10, a cytokine associated with immune regulation, significantly increased in all three pain phenotypes (*P* < 0.05), with the largest increase observed in the non-inflammatory pain phenotype, reflecting an adaptive immune response.

In the non-inflammatory pain phenotype, interventions were focused primarily on psychosocial and neuromuscular mechanisms. As a result, cytokines such as IL-6 and IL-17A were not targeted for modulation and therefore were not analyzed for this phenotype (Figure 2). These findings reinforce that cytokine modulation is more relevant in the inflammatory and mixed phenotypes, while psychosocial mechanisms dominate in non-inflammatory pain.

### Structural equation modeling (SEM) of mechanistic pathways

To further elucidate the interrelationships between biological and psychosocial mechanisms of pain, SEM was performed. The hypothesized model incorporated four latent constructs: inflammatory activity (CRP, ESR, IL-6, TNF-α, Th17/Treg ratio), psychosocial distress (HADS-A, HADS-D, PSQI, PCS), central sensitization (Substance P, CGRP), and patient-reported outcomes (VAS, HAQ, SF-36). The model demonstrated acceptable goodness-of-fit (χ^2^/df = 2.14, RMSEA = 0.053, CFI = 0.94, TLI = 0.92), indicating that the proposed mechanistic structure adequately represented the observed data.

As shown in Table 6, inflammatory activity exerted a significant direct positive effect on central sensitization (β = 0.43, *p* < 0.001), suggesting that higher systemic inflammation was associated with greater activation of pain amplification pathways. Inflammatory activity also showed an indirect effect on patient-reported outcomes mediated through psychosocial distress (β = 0.22, *p* = 0.002), indicating that inflammation influenced pain severity and functional impairment partly by increasing emotional and cognitive burden.

**TABLE 6:** Standardized path coefficients in the structural equation model.

Pathway	Standardized β	*p*-value	Interpretation
Inflammatory activity → central sensitization	0.43	< 0.001	Stronger inflammation increases neuropeptide activity
Inflammatory activity → psychosocial distress	0.27	0.004	Inflammation correlates with anxiety, depression, poor sleep
Psychosocial distress → patient-reported outcomes	−0.51	< 0.001	Greater distress worsens pain and QoL
Central sensitization → patient-reported outcomes	0.33	< 0.001	Neuropeptide activation increases perceived pain
Inflammatory activity → patient-reported outcomes (direct)	0.19	0.018	Residual direct biological effect
Indirect inflammation → psychosocial → outcomes	0.22	0.002	Mediated pathway through distress
Total variance explained (R^2^)	0.72	−	Model explains 72% of variability in outcomes

Psychosocial distress had a strong direct negative impact on patient-reported outcomes (β = −0.51, *p* < 0.001), demonstrating that anxiety, depression, sleep disturbance, and catastrophizing were major determinants of worse pain perception and functional limitation, independent of inflammatory status. Central sensitization contributed additional explanatory value to patient-reported outcomes, supporting its role as an intermediate mechanism linking biological inflammation and subjective pain experience.

The indirect pathway from inflammatory activity to patient-reported outcomes via psychosocial distress accounted for approximately 18% of the total variance in pain scores, highlighting the importance of non-inflammatory pathways in amplifying pain burden. Overall, the final SEM accounted for 72% of the variance in patient-reported outcomes, providing quantitative evidence that RA pain is driven by an integrated network of inflammatory, neurobiological, and psychosocial mechanisms rather than by a single dominant pathway.

## Discussion

The present study demonstrates that rheumatoid arthritis (RA) pain arises from the interaction of inflammatory activity, psychosocial distress, and central sensitization, and that these mechanisms can be effectively integrated within the Nursing Science Precision Health (NSPH) framework (12). By combining clinical indicators, biomarker profiles, and psychosocial assessments, our findings indicate that pain is not solely driven by inflammation but is substantially shaped by emotional and behavioral factors. Structural equation modeling (SEM) further supports this multidimensional mechanism, showing that inflammatory activity influences pain both directly and indirectly through psychosocial distress and neuropeptide-mediated sensitization. The strong effect of psychosocial distress on patient-reported outcomes suggests that psychological regulation is mechanistically involved in pain perception and functional limitation, rather than serving only a supportive role.

A major contribution of this study is the application of pain phenotyping to guide targeted interventions. Patients categorized into inflammatory, non-inflammatory, and mixed phenotypes exhibited distinct biological and psychosocial characteristics, which translated into different treatment responses. In the inflammatory phenotype, reductions in CRP, ESR, and pro-inflammatory cytokines following pharmacological therapy confirm that inflammation remains a key driver of nociceptive pain, consistent with prior studies on DMARDs and biologics (26, 27). These findings reinforce the importance of early and adequate inflammatory control to reduce pain and prevent disease progression (28). Notably, decreases in IL-6, IL-17A, and TNF-α, together with increased IL-10 and improved Th17/Treg balance, suggest that phenotype-based interventions may restore immune homeostasis beyond conventional inflammatory markers (29), which highlights the value of incorporating broader immunological indicators into clinical monitoring.

Importantly, this study also emphasizes the clinical relevance of non-inflammatory pain, which is frequently under-recognized in RA management (15, 30). Patients in this subgroup showed higher levels of anxiety, depression, and sleep disturbance despite lower inflammatory activity, indicating that central sensitization and psychosocial factors are dominant drivers of pain. Improvements in fatigue, catastrophizing, and quality of life following psychosocial interventions further support the effectiveness of targeting these pathways. These findings are consistent with previous evidence linking psychological distress and maladaptive coping to increased pain perception (31). Clinically, this underscores the need to complement pharmacological treatment with structured psychological and behavioral interventions, particularly for patients with persistent pain despite controlled inflammation.

The inclusion of biomarker analysis provides additional mechanistic insight. Reductions in IL-6 and TNF-α following intervention suggest that these cytokines contribute to pain sensitization beyond their role in inflammation, including in mixed or non-inflammatory phenotypes (32). Concurrent increases in IL-10 and improvements in oxidative stress markers (MDA, SOD, GSH-Px) indicate a shift toward anti-inflammatory and antioxidative states. Decreases in Substance P and CGRP support reduced central sensitization, while normalization of cortisol and ACTH suggests partial recovery of neuroendocrine function (33). Together, these findings highlight that effective pain management requires coordinated modulation of immune, neural, and endocrine pathways. The observed reduction in anti-CCP antibodies also suggests that phenotype-based interventions may influence autoimmune activity (34, 35), although this requires confirmation in longitudinal studies. Another important implication is the potential of pain phenotyping to predict treatment response. Inflammatory markers such as CRP and ESR were associated with improvement in the inflammatory phenotype, whereas psychosocial variables were more predictive in the non-inflammatory group (36). Additionally, ROC analysis identified IL-6 and FACIT-F as useful predictors of severe pain, indicating that combining biological and psychosocial indicators improves patient stratification. This multidimensional approach may help clinicians identify patients at risk of persistent pain and guide more targeted interventions.

From a practical standpoint, the NSPH model offers a structured approach to implementing these findings in real-world settings. The model’s emphasis on accurate symptom measurement, phenotypic analysis, and biomarker discovery aligns with the growing trend toward personalized medicine (12). For healthcare providers, adopting this framework could facilitate more precise and effective care delivery, reducing trial-and-error approaches and enhancing patient satisfaction. For policymakers, the study provides a rationale for expanding support for multidisciplinary pain management programs, including access to psychological counseling, dietary interventions, and complementary therapies like acupuncture. The findings also have implications for health education and patient empowerment. Many RA patients, particularly those with non-inflammatory pain, may not fully understand the multifactorial nature of their symptoms or the potential benefits of non-pharmacological therapies. Incorporating pain phenotyping into patient education programs could improve awareness and engagement, helping patients make informed decisions about their care. For instance, patients with non-inflammatory pain may benefit from targeted education about the role of stress management and sleep hygiene in pain modulation. Similarly, providing dietary counseling tailored to individual phenotypes could encourage adherence to anti-inflammatory diets, further enhancing outcomes.

Operationalizing the NSPH model in RA care enables nurses to translate multidimensional assessment data into individualized pain management strategies. Within this model, nurses play a pivotal role across four domains: (1) precise symptom measurement, employing integrated pain, psychological, and sleep assessment tools (VAS, GPS, HADS, PSQI) to capture the multifaceted nature of pain; (2) phenotype-based assessment, synthesizing laboratory biomarkers (CRP, ESR, IL-6, TNF-α, Th17/Treg ratio) with psychosocial indicators to differentiate inflammatory, non-inflammatory, and mixed pain patterns; (3) personalized intervention planning, including medication adherence guidance, cognitive-behavioral and mindfulness-based therapies, and lifestyle optimization focusing on sleep and nutrition; and (4) continuous outcome monitoring, where nursing teams integrate longitudinal pain and quality-of-life data to refine interventions in real time. Such integration transforms nursing practice from a supportive to a decision-making role within interdisciplinary RA management, allowing nurses to bridge biological, behavioral, and environmental dimensions of chronic pain. Consistent with previous findings (12–14), precision nursing facilitates early symptom detection, psychosocial support, and adaptive care planning. By operationalizing these processes, nurses can contribute to data-driven, phenotype-specific pain management, thereby enhancing both patient outcomes and professional autonomy in rheumatology care settings.

Nevertheless, the current study has several limitations. First, its retrospective design limits the ability to establish causal relationships between pain phenotypes, biomarkers, and psychosocial factors. Longitudinal or interventional studies are needed to verify the dynamic interaction among inflammatory and non-inflammatory mechanisms of pain in rheumatoid arthritis (RA). Second, all participants were recruited from a single regional cohort, which may restrict the generalizability of findings to broader RA populations with diverse demographic or clinical profiles. Third, biomarker assessment focused primarily on cytokine and immune parameters. Future studies could incorporate multi-omics analyses, including metabolomic and neuroimaging indicators, to more comprehensively characterize pain phenotypes. Finally, although this study applied the NSPH model conceptually, practical implementation in routine nursing workflows requires further multicenter validation and development of digital assessment tools to enhance precision and scalability. Further comprehensive work should aim to expand these findings through longitudinal, technology-supported nursing interventions to optimize individualized pain management.

In conclusion, our study underscores the potential of the NSPH model in transforming pain management for RA. By tailoring interventions to individual pain phenotypes and addressing both inflammatory and non-inflammatory mechanisms, this approach offers a pathway to more effective, personalized care. Beyond RA, the findings highlight the broader applicability of precision health principles, providing a foundation for advancing pain management across a range of chronic conditions. Future efforts should focus on optimizing the implementation of this model, ensuring its scalability and sustainability in diverse healthcare settings.

## Data Availability

The raw data supporting the conclusions of this article will be made available by the authors, without undue reservation.
